# Repair: The Interface Between Interaction and Cognition

**DOI:** 10.1111/tops.12339

**Published:** 2018-05-10

**Authors:** Saul Albert, J. P. de Ruiter

**Affiliations:** ^1^ Department of Electronic Engineering and Computer Science Queen Mary University of London; ^2^ Department of Psychology Tufts University; ^3^ Department of Computer Science and Department of Psychology Tufts University

**Keywords:** Repair, Interaction, Conversation analysis, Conversation, Communication, Understanding, Dialog, Ethnomethodology

## Abstract

Conversational repair is the process people use to detect and resolve problems of speaking, hearing, and understanding. Through repair, participants in social interaction display how they establish and maintain communication and mutual understanding. We argue that repair provides a crucial theoretical interface for research between diverse approaches to studying human interaction. We provide an overview of conversation analytic findings about repair in order to encourage further cross‐disciplinary research involving both detailed inductive inquiry and more theory‐driven experimental approaches. We outline CA's main typologies of repair and its methodological rationale, and we provide transcripts and examples that readers can explore for themselves using open data from online corpora. Since participants in interaction use repair to deal with problems as they emerge at the surface level of talk, we conclude that repair can be a point of convergence for studying mis/communication from multiple methodological perspectives.

## Abstract 2

Albert and De Ruiter provide an introduction to the Conversation Analytic approach to ‘repair’: the ways in which people detect and deal with troubles in speaking, hearing and understanding in conversation. They explain the basic turn‐taking structures involved, provide examples, explain recent developments in the field and highlight some important points of contact and contrast with work in the Cognitive Sciences.

## 1 Introduction

Imagine a world without methods to deal with miscommunication. In this world, if some coordination problem emerged in the course of a joint activity—building a very tall tower, for example—it might take so long to fix that in the process of resolving one problem, more problems would have time to emerge. Nothing that required any degree of mutual understanding and coordination would be possible. Although it may sometimes feel as though we live in this confused and uncoordinated world, the evidence of our ability to engage in structured collaborative activities is all around us. Our highly complex systems for living, working and sustaining relationships with each other clearly demonstrate the effectiveness of human communication. We must therefore have practical methods for maintaining joint coordination and understanding between individuals as a basic requirement of interaction. These methods enable us to fix problems more quickly than they can emerge and multiply. The field of conversation analysis (CA) calls this area of research “repair.” The purpose of this article is to outline what has been discovered about repair and to provide a primer featuring detailed analytic characterizations of different types of repair for researchers in the cognitive sciences. Within CA, studying repair involves observing and describing all the ways people work to identify and resolve “troubles of speaking, hearing and understanding” (Schegloff, Jefferson, & Sacks, 1977, p. 361) as they emerge in interaction. In cognitive science and psychology, on the other hand, dialog research tends to theorize about mental representations and cognitive architectures that underpin shared understanding, and then work out how to test those processes experimentally (Clark & Brennan, 1991). These approaches are often seen as fundamentally incompatible, and CA's tendency to avoid theorizing about mental states or to use informational measures of shared understanding has sometimes been mistaken for a behaviorist rejection of subjectivity in general (Maynard & Clayman, 2003). This paper aims to dispel that view by showing how CA's methodological rationale is designed to remain focused on practical interactional problems at the surface of talk, and how people work to maintain intersubjectivity on a moment‐by‐moment basis. This approach makes CA an especially useful approach to miscommunication. The surface‐level appearance of miscommunication in dialog and the ways participants work to fix it may constitute the most secure, observable evidence of communicative success available to participants in an interaction (Gregoromichelaki et al., 2011). The central argument of this paper is that the study of repair can therefore provide an interface for cross‐disciplinary research on communication between conversation analysis and other approaches to the study of human interaction.

### 1.1 Miscommunication and shared understanding

“Miscommunication” is a paradoxical concept from a CA perspective because it is the very methods people use to repair problems in interaction that provide CA (and participants in interaction) with the best evidence that communication has been successfully achieved. According to CA, mutual understanding is not achieved by having sufficiently similar mental representations about the world, as some dialog researchers from the cognitive sciences and psycholinguistics have assumed (Anderson et al., 1991; Pickering & Garrod, 2004). Rather, understanding is a situation where people treat whatever they can observe as sufficient—for all practical intents and purposes (Garfinkel, 1967, pp. 7–9)—to progress with whatever they are doing together. Macbeth (2011) calls CA's notion of understanding “an exquisitely local affair”: because CA treats the progressive, turn‐by‐turn production of talk as standing on behalf of a kind of contingently shared understanding. This form of understanding is “local” to each moment in each specific situation. If it later transpires that people have come away with different accounts of what happened or what was understood, their episode of communication was still conducted successfully enough for the participants at the specific moment at which it occurred. Once participants have recognized a sequence of interaction as complete by closing it off (Schegloff & Sacks, 1973), another interaction to deal with ostensible misunderstandings would constitute a new local episode of interaction. As participants in conversation progress from one turn at talk to the next, they treat the *absence of evidence of misunderstanding* as sufficient to allow the interaction to progress. CA describes this as a principle of “progressivity” (Robinson, 2014; Zama & Robinson, 2016) whereby if the interaction progresses without ostensible misunderstandings being flagged up, participants will proceed as though shared understanding has been achieved.

### 1.2 CA's approach to misunderstanding

This minimal notion of shared understanding as progressivity (Hindmarsh, Reynolds, & Dunne, 2011) is an intentional constraint on CA's analytical repertoire. The study of repair focuses analysis on the “broken surface of talk” (Jefferson, 2017, p. 132) where analytical evidence is also manifestly available to participants in interaction, rather than relying on factors beyond the current situation. This sets aside questions of context and reference resolution, which are central to experimental and computational approaches to semantics and pragmatics in dialog (Ginzburg, Fernández, & Schlangen, 2014; Healey, Purver, King, Ginzburg, & Mills, 2003; Hough & Purver, 2017).^1^ Streeck (2010) describes this as a “maxim of contextual parsimony”: to concentrate on each situation and see what its details can reveal about the methods participants use to resolve interactional trouble in situ (Lynch, 2000, p. 522). This methodological commitment to induction and observation over deduction and experimentation (De Ruiter & Albert, 2017) has yielded detailed descriptions that are useful to, although epistemologically distinct from, more theory‐driven research in related fields (Nishizaka, 2015). CA's approach to misunderstanding is similarly minimal. Since abstract concepts such as “understanding” are not usually discussed or verified explicitly during interaction, participants simply keep going and rely on repair as a method to fix any problems as and when they occur. Grace Hopper's famous heuristic for dealing with bureaucracy: that “it's easier to ask forgiveness than to get permission” (Hamblen, 1986) is a good metaphor for repair as a process. Repair provides a highly efficient method for “restoring progressivity” (Kent & Kendrick, 2016) only after it has evidently broken down, eliminating the cumbersome work of preempting all imaginable problems during the planning of an utterance (Clark & Marshall, 1981, pp. 15–16), or the infinite regress of having to separately check for evidence of understanding after each turn (Clark & Brennan, 1991, p. 133). Instead, CA tracks how people continue to participate in the flow of turn‐by‐turn talk and embodied action unless and until they stop to address some kind of problem that requires repair (Goodwin, 2000; Hindmarsh et al., 2011). This analytic assumption reflects how the participants treat the ongoing progress of talk as sufficient evidence of shared understanding to simply proceed with the current interaction.

A surface‐level account of shared understanding allows analysts to explore *misunderstandings* by focusing on how people expose and resolve interactional problems as they emerge. CA's descriptive, inductive, and empirical use of detailed transcription and analysis of audiovisual recordings of interaction (Haddington, Mondada, & Nevile, 2013, p. 47) creates procedural descriptions based on observations of how people use systems of turn‐taking, repair, and other components of a broader “technology of conversation” (Sacks, 1984). Findings are usually reported using line‐by‐line analysis of interaction rather than statistical aggregates or, until relatively recently, the kinds of quantitative analyses that are common in many areas of interaction research (Stivers, 2015). This is partly because CA has traditionally tried to avoid prematurely operationalizing and coding features for quantification (Levinson, 1983, 294) where the interactional dynamics of the phenomenon in question might not yet be fully understood.^2^ Studying repair in detail involves exploring the sequential organization of contributions to dialog between participants in the “repair space” (Schegloff et al., 1977, p. 375): the time between the moment at which the speaker or recipient flags up a problem and the problem's resolution. “Resolution” here is marked by the resumption of progressivity, which functions, in practice, as an assumption of shared understanding. Researchers can then analyze the many ways people can suspend, repair, and resume progressivity. Analyses of repair build on the famous turn‐taking system for conversation first described by Sacks, Schegloff, and Jefferson (1974), which provides a clear framework for describing orderly contributions to conversation in sequential terms. This framework has inspired many attempts both to generalize (Stivers et al., 2009) and refute (Heldner & Edlund, 2010) CA's turn‐taking system experimentally. Sequences of turns at talk are built from clausal, phrasal, or sentential “turn constructional units” (TCUs), each of which ends by producing a “transition relevance place” (TRP), where the turn is apparently complete and then speaker change may (optionally) occur in the next turn. The system also describes how participants recursively allocate speaker and recipient roles at each TRP and manage this issue of spontaneous mutual coordination turn‐by‐turn. This turn‐taking “machinery” (Schegloff & Sacks, 1973) highlights problems of speaking, hearing, and understanding on the surface of interaction, where hitches, disfluencies, or halts reveal aspects of whatever problem may need to be resolved in order for progressivity to resume.

### 1.3 Turn‐taking and repair

Although a full description of CA's turn‐taking system is beyond the scope of this article,^3^ its broad structure can be summarized in terms of how people organize repair. Extract 1 shows some examples of consecutive sequences of action: a greeting sequence and two question/answer (“how are you”) sequences. After each turn in the following two examples, the turn number (T), current position/s (P) in a sequence of action, and the broad action “type” is provided in double parentheses. Some examples here are from the literature but most are drawn from the AudioBNC corpus (Coleman, Baghai‐Ravary, Pybus, & Grau, 2012), which enables linking to extracts within their audio files. This can provide researchers unfamiliar with conversational data a clear sense of the speed and accuracy with which these sequences are organized.^4^

**Table:** 

**Extract 1** Three “paired” action sequences/CABNC 021A‐C0897X0385XX‐AAZZP0		
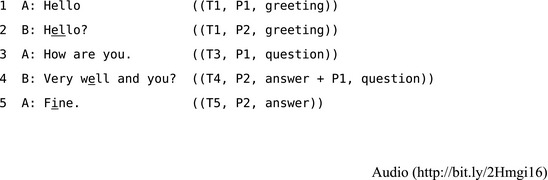		

Note that a turn may involve multiple actions (Schegloff, 2007, pp. 75–76), for example, turn 4 in Extract 1 provides the answer “Very well” to the initial “how are you” question in turn 3, and also asks an anaphoric version of this question with “and you?” in the same turn.

### 1.4 Approaches to misunderstanding in cognitive science and CA

Extract 2 shows how two question/answer sequences can be “nested,” one inside the other. This schematic example highlights the structural organization of sequences between turns. The numbering in double parentheses shows how turns and sequences are structurally independent features of the turn‐taking system. Turn‐position is organized serially, whereas each action is numbered in relation to its sequential relevance, usually in relation to a grammatically or syntactically fitted and correspondingly “typed” prior action.

**Table:** 

**Extract 2** A set of “nested” question/answer pairs cited in Levinson (1983, p. 304)		
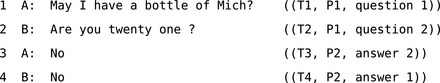		

While schematic examples such as Extract 2 are useful for introductory explanations, it is important to be able to hear how apparently effortlessly these sequences can be organized on the fly. The following extracts use transcription conventions developed by Gail Jefferson (Hepburn & Bolden, 2012) to highlight details such as sound stretches, emphasis, and overlap (see Appendix A for a key to these symbols). In a more intricately transcribed example in Extract 3, Patricia asks Beth to remind her about the meal her husband made for her on the previous evening.

**Table:** 

**Extract 3** “Nested,” paired action sequences/CABNC 021A‐C0897X0615XX‐AAZZP0		
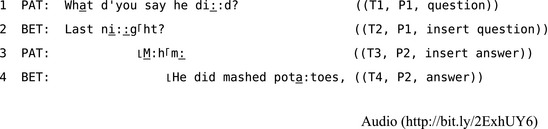		

In line 2, Beth checks whether Patricia has asked about last night's dinner, and Patricia answers (minimally) in line 3. Once this issue is resolved, Beth proceeds with her answer to the initial question in line 4. These kinds of “insert expansions” (Schegloff, 2007, pp. 100–106) are often used to repair ambiguities or trouble with some prior turn. More generally, CA's findings about repair rely on tracking this flexible structure of turn and sequence because when problems emerge with speaking, hearing, or understanding, then the work people do to fix the problem usually requires them to reorganize the order of turns and sequences. The first position at which repair is possible occurs before a sequence emerges; that is, in the first turn, where speakers can monitor their own talk and deal with any problems by initiating repair. This involves a speaker halting their own turn at talk and repairing it before continuing—a process termed “self‐repair” both in CA (Schegloff et al., 1977) and in the cognitive sciences more broadly (Levelt, 1983).

The end of this first turn occurs after the speaker reaches a point of possible completion (a TRP) where another speaker may take the next turn. At this point, if a speaker initiates repair, they no longer just halt the progress of a turn, they halt the progress of a sequence (Kitzinger, 2012). Therefore, beyond “same‐TCU repair” by the speaker of the trouble‐source, the turn‐taking system continually provides further sequential “positions” where participants may initiate and complete repair. These positions have differing sequential characteristics, routine uses, and procedural dynamics. For example, participants in dialog routinely produce paired sequences of action such as greetings followed by responsive greetings, or initial invitations followed by subsequent acceptances or rejections (Sacks, 1987). The two‐part structure of these action sequences means that first, second, and third position are particularly prominent sequential positions for the initiation and production of repair.^5^

1. *Self‐initiated repair in same‐TCU* is initiated by the current speaker in their own TCU (e.g., Extract 4a) or in the transition space before anyone else has a chance to take a turn. It reveals some element of the speaker's turn as a trouble‐source by resolving it.
2. *Other‐initiated repair* (e.g., Extract 4b) often occurs in the next slot available as soon as another speaker takes a turn in a given sequence. When repair is initiated by a recipient, it treats the initial speaker's talk as a trouble‐source and creates an opportunity for either the recipient or the initial speaker to provide the repair solution.
3. *Third position repair* (e.g., Extract 5) is initiated by the speaker (who produced the first turn) as a third “move” in a sequence of actions when the recipient's response reveals their misunderstandings or mishearings of the trouble‐source turn.

**Table:** 

**Extract 4** Two simplified examples from (Schegloff et al., 1977, pp. 36–37)	
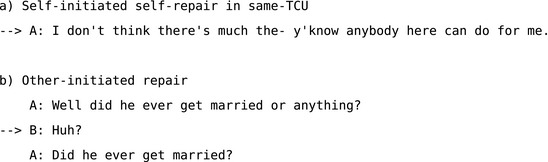	

**Table:** 

**Extract 5** A simplified 3rd position repair from (Schegloff, 1992, p. 1303)	
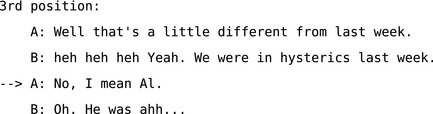	

These prominent types and positions for repair^6^ provide a flexible framework for being very specific when describing the order of events in a repair operation. By specifying what happens and in which order during repair, researchers can trace how the detailed sequential structure of repair points to participants’ displays of shared (or non‐shared) understanding. For example, the use of “huh” in Extract 4b is a very different method for locating the ostensible source of “trouble” in the prior turn than the use of “No, I mean Al” in extract 5. The choices participants make about which repair methods to use and how they use them can reveal a lot about their own analysis of the state of their interaction as it unfolds.

### 1.5 How repair points to interactional rather than informational states

Because repair deals with *displays* of understanding and how people make choices about what they display, it is crucial to note the distinction Jefferson (1987) makes between repair and forms of exposed or embedded correction in dialog. Exposed corrections are done “on the record” because they explicitly delay or disrupt progressivity. Embedded corrections, on the other hand, are smuggled through, officially unnoticed, within the flow of ongoing interaction. This distinction clarifies how repair works primarily as a surface‐level method for restoring progressivity in dialog rather than as a means of achieving identical states of information between participants.^7^ For example, in Extract 6 a salesman provides an embedded correction of the customer's use of the term “wales” in line 1.

**Table:** 

**Extract 6** (15a [GJ:FN]) from Jefferson (1987, p. 93)	
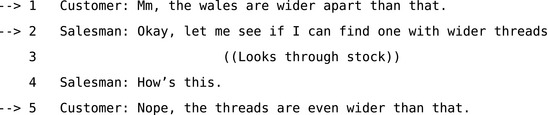	

Despite apparent differences in their knowledge of terminology, there is no obvious interactional problem here because there is no halt in progressivity. Participants converge on using the term “threads” without halting progressivity. Even where there is no evident difference in the information available to participants, repair may deal with more diffuse problems such as impropriety or transgressions of social norms. For example, in Extract 7, when the mother grants the child's request after line 5, the problem is revealed to be an issue of politeness—or at least the child's omission of “please” in the initial version of the turn.

**Table:** 

**Extract 7** Extract 22 [Johnstone:14:068] from Drew (1997, p. 95)		
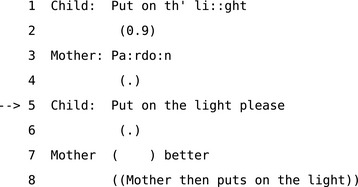	

The distinction between exposed and embedded correction turns on their impact on progressivity because the latter allows for correction without making an issue of a problem and holding up the interaction. If a participant were to stop in the midst of a conversation and say “there has been a misunderstanding, let's sort it out,” this is not only obstructive, it may even constitute a different activity and context entirely. For example, this could be one way for participants to show that they are now engaged in the activity and context of “having an argument”. Whatever participants may know in common—or not—and whatever their psychological motivations may be for a repair, surface‐level analysis can only show how they choose to expose these issues to each other through interaction. This means that the psychological motivations for repair may not be accessible for analysts unless, as sometimes happens, the participants in the interaction themselves go on to address their motivations explicitly. These kinds of analytic constraints are not necessarily a problem, since whether a repair addresses breakdowns in process, reference, or some contextual issue such as inappropriateness or unexpectedness people can initiate repair and resolve any kind of trouble without necessarily specifying motivations or causes. This points to one of the reasons that repair is such a useful and efficient mechanism. Since repair only has to achieve *apparent* understanding and only has to do that sufficiently to resume progress, it is flexible and generic enough to deal with many kinds of interactional problem.

## 2 An overview of canonical types of repair

Repair seems to be a priority activity since when someone initiates repair at any point in an interaction, participants suspend progress and work to deal with the problem until it is resolved (Sacks et al., 1974, p. 720). Where interaction is delayed or derailed in this way the work people do to fix the problem reveals how they have analyzed and understood it. This provides an analytic foothold from which to explore repair and what happens just before and after it occurs. Since a repair has to be initiated by one or another party, analysts tend to start by asking who is accountable–in the sense of responsible (Robinson, 2016, p. 12)–for identifying a problem in interaction by initiating repair. The next question, then, is which party takes responsibility for solving the problem by offering a “repair solution.” Schegloff (1987) notes that there does not appear to be any systematic relationship between the type of trouble‐source (whether hearing, speaking, understanding, or some kind of social impropriety) and the form of repair chosen to resolve it. People may also use repair to design social actions such as invitations, questions, or compliments in ways that use repair as a format for a specific kind of action rather than as a way to fix something. For example, inviting someone to dinner while hesitating, repairing, or otherwise wavering on the details may reveal to recipients that the relevant, expected response is to refuse or defer the invitation rather than to accept it.^8^ Repair can therefore function as an “interactional resource” (Jefferson, 1974) to formulate delicate or potentially problematic actions such as joking or teasing (Schegloff, 1987) in ways that modulate the social accountability of the speaker for whatever is being said or done. One of the most crucial questions to start with when studying repair, then, is which party to an interaction identifies and exposes the problem, and which resolves it and thus allows the interaction to continue.

### 2.1 “Self” and “other” initiation and repair

There are four key analytic distinctions based on which party initiates a repair which party resolves it. “Self‐initiated” repairs are initiated by the producer of a trouble‐source (“self”).

“Other‐initiated” repairs are initiated by the recipient of the trouble‐source (“other”). The corollary distinction is between which party—“self” or “other”—completes a repair by resolving the trouble. Since dealing with trouble in speaking, hearing or understanding may involve multiple parties, “self” and “other” here refer to shifting participation roles which may be distributed between multiple persons involved in a conversation (Bolden, 2013).

“Self‐repair” is completed by the producer of the trouble‐source, and “other‐repair” by its recipient. This provides a basic four‐way taxonomy: self‐initiated self‐repair (SISR), self‐initiated other‐repair (SIOR), other‐initiated self‐repair (OISR), and other‐initiated other‐repair (OIOR). Some of the basic empirical questions we can ask about repair involve using these distinctions to explore interactions where there seems to be some trouble, then seeing who first registers it by initiating repair, who solves it, and what this means for the ordering or priority of repair operations. For example, early research on repair focused on exploring why and how self‐initiated repairs seemed so prevalent in everyday talk relative to other‐initiated repairs (Schegloff et al., 1977). The prevalence of self‐initiated self‐repairs in the same TCU makes sense intuitively because these repair‐initiations occur at the first opportunity for repair. Repair also deals with problems of hearing and understanding that can only be exposed after the first turn by recipients’ subsequent responses. This structural consequence of the turn‐taking system may also contribute to the apparent prevalence of self‐initiated self‐repairs relative to other forms. CA uses the term “structural preference” to describe the way these sequential properties result in an emergent distribution of types of repair. Note that the terms “preference” and “dispreference” here are not used to ascribe any subjective or psychological likes or dislikes to persons involved in an interaction. Rather, they describe how sequential structures tend (e.g., in the case of self‐initiated self‐repair) to lead to the prevalence of one or another solution to a routine kind of interactional problem.

### 2.2 Self‐initiated self‐repair

The most structurally preferred form of repair is self‐initiated self‐repair in the same TCU (Schegloff et al., 1977) since self‐repair is initiated by a speaker monitoring and adapting their own turn before recipients have a chance to respond. Even at a point of possible turn‐completion at a TRP, speakers may still self‐initiate repair on their just‐prior turn in the “transition space” before recipients have used the opportunity to take the next turn (Schegloff, 1979, p. 269).^9^ For example, in Extract 8, where Pam and Lyn are overlooking and describing groups of performers from a balcony above them, Pam does several self‐initiated self‐repairs in the course of producing her description.

**Table:** 

**Extract 8** TATM2012_P‐L_0709 (https://git.io/vPb6G)	
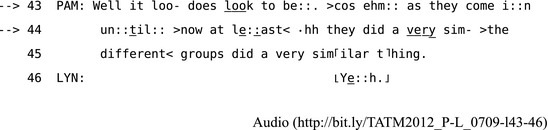	

**Figure 1:**
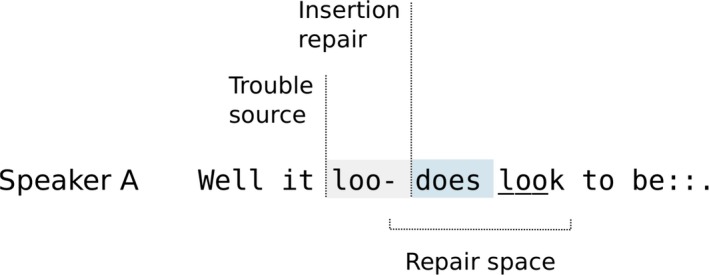
A schematic of the first same‐turn self‐initiated self‐repair Extract 8.

Pam opens the repair space by self‐initiating repair on her own turn several times. First, she cuts off at two points: “loo” in line 43 and “sim‐” in line 44. Then, she self‐repairs using an *insertion repair* procedure (Wilkinson & Weatherall, 2011) that adds several new elements: respectively “does” and “the different groups did a very” to her turn at the two cut‐off points before closing the repair space and resuming progressivity. Pam marks the resumption by repeating the previously cut‐off elements “look” and “similar” in full, and in each case these repeats close the repair space. In Fig. 1, the structure of this type of “insert repair” procedure is illustrated using a schematic (that follows the style used by Dingemanse et al. (2015).

Through this kind of insertion repair procedure a speaker can cut off a turn in progress and repair it, often by adding more specific or intensified items (Wilkinson & Weatherall, 2011). This is one of a range of self–initiated self‐repair procedures that include methods for replacement, insertion, or deletion of items and many other modifications of the ordering, format, or sequential structure of the repairable turn (Schegloff, 2013).

### 2.3 Self‐initiated other‐repair

A common form of self‐initiated other‐repair is a “search,” where the speaker (self) cuts off, hesitates, or becomes disfluent in the midst of a TCU, demonstrating that they are having a problem with continuing. This provides an opportunity for the recipient (other) to do the repair. In Extract 9, Jamie is struggling to recall the full name of an author, but he provides Anne with enough clues for her to produce the repair solution.

**Table:** 

**Extract 9** CABNC 021A‐C0897X0702XX‐AAZZP0		
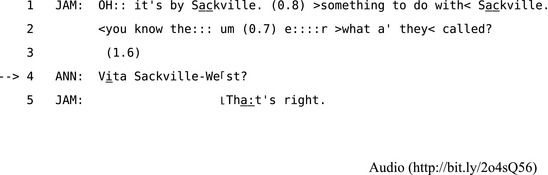	

**Figure 2:**
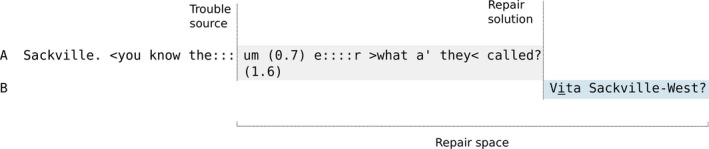
A schematic of a the self‐initiated other‐repair provided in line 3 of Extract 9.

Jamie's oh‐prefaced turn at the beginning of Extract 9 marks his having just now remembered something (Bolden, 2006; Heritage, 1984a); in this case, the name “Sackville.” After a pause during which he receives no apparent uptake from Anne he moves toward a search for the name. First, he equivocates over the adequacy of the name he has already produced by adding that it is “>something to do with< Sackville.” Jamie's “you know,” first suggests that Anne might know the author, and then he produces a long sound stretch on “the:::” and pauses at “um.” From this point in the TCU it is clear that Jamie's disfluency constitutes a search. He finally explicitly requests that Anne tells him “what a” they< called?” until finally Anne provides the solution, which Jamie quickly confirms in overlap. Once this search is resolved, they move on. The schematic in Fig. 2 shows how this kind of “search” repair space can be opened using sound‐stretches, long pauses, and may include hints and prompts such as “whatchamacallit” or “whatsisname” or partial solutions that help recipients to provide a candidate repair (Lerner, 1996, pp. 262–264).^10^ However, without these kinds of projectable hints and clues speakers may also produce “self‐directed” searches (Lerner, 2013) that suspend the sequence until they can resolve the problem themselves as a form of SISR. In general, searching may not always fit into categories of SISR or SIOR when gaze organization is taken into account (M. Goodwin, 1980; M. Goodwin & Goodwin, 1986): The embodied construction of searches within turns and smaller “sub‐units” of TCUs (Iwasaki, 2011) may be designedly ambiguous in ways that can sustain the opportunity for either self‐ or other‐repair.

### 2.4 Self‐initiated self‐repair in third position

Third position repair deals with a trouble‐source exposed by a recipient's response to a prior turn. Schegloff (1992) describes third position repair as the “last structurally provided defense of intersubjectivity in conversation” because it is at this point in a sequence that the problems revealed to the speaker by the recipient's response can—if unchecked—lead to a breakdown in mutual understanding that escapes beyond the local structure of a sequence.^11^ In Extract 10, for example, Claire is watching television as her mother Helen has finished cooking. When her mother asks Claire to “switch that off,” Claire's response shows Helen that her daughter has misunderstood.

**Table:** 

**Extract 10** CABNC KCD 004819	
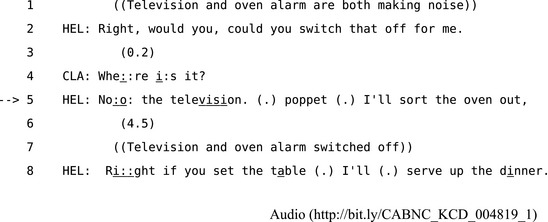	

Claire's question at line 4 also reveals precisely how she misunderstood Helen's request as a request to turn off the oven alarm rather than the television. In line 5 Helen does a third position repair by specifying that she meant the television and saying she will switch off the oven herself. The repair clearly succeeds since they complete their respective tasks and move on to setting the table and serving dinner. This kind of situation—where a recipient's response reveals a referential ambiguity—is often resolved by a third position repair. For example, in Extract 11, Evelyn asks her husband Arthur about his newspaper in a way that leaves it ambiguous as to whether she means the newspaper as a whole or the specific passage he is currently reading.

**Table:** 

**Extract 11** CABNC KBB 049008	
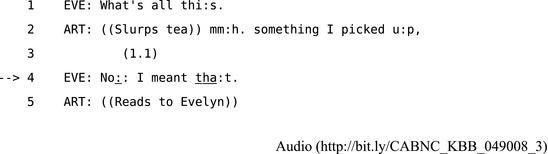	

**Figure 3:**
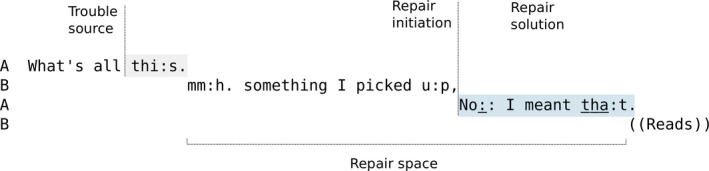
The sequential structure of the third position repair in Extract 11.

Arthur's response at line 2 treats Evelyn's question as an inquiry about where he got his newspaper. Then in third position at line 4 Evelyn repairs this misunderstanding and Arthur responds by reading out the story. The structure of this third position repair can be illustrated particularly clearly in Fig. 3 because Evelyn's repair is able to resolve the relatively limited problem of reference within a single sequence.

### 2.5 Other‐initiated self‐repair

Other‐initiated self‐repair (OISR) is a repair operation initiated by any party other than the speaker of the trouble‐source (Schegloff et al., 1977) where the repair is then completed by the speaker. Other‐initiated repairs in general suspend whichever sequence they arise in and constitute a “nested” sequence of their own (Clift, 2016, p. 247). Other‐repair is very often initiated in the turn immediately following the trouble‐source (Schegloff, 2000, pp. 210–211). For this reason common OIR devices such as “huh?”—which are almost always produced in second position (Enfield et al., 2013)—were initially referred to as “next‐turn repair initiators” or “NTRIs” (Schegloff, 1995, p. lvi), until conversation analysts found that other‐initiations of repair also occur beyond the next turn (Schegloff, 2000) and began finding more detailed ways in which the “next turn” position could be used for repair (Drew, 1997). This term has now been deprecated following the discovery of a wide variety of other OIR devices and methods that require more detailed descriptions. For example, some OIR devices such as “what?” or “pardon?” are language‐specific, whereas the repair initiator “huh?” appears to be so consistent and similarly pronounced across language groups that Dingemanse, Torreira, and Enfield (2013) describe it as a “universal word.” More structural, language‐independent methods for other‐initiation (Schegloff et al., 1977) and non‐vocal methods for initiating repair (Manrique, 2016; Manrique & Enfield, 2015) rely on their position in the next turn relative to the trouble‐source to identify it as the “repairable.” Other methods can identify the repairable using a range of less positionally sensitive devices and these methods are often grouped in terms of their relative “power” to locate and resolve the trouble‐source (Schegloff et al., 1977, p. 369). “Open class repair initiators” (Drew, 1997) such as “huh?”, “what?”, or “sorry?” are less “powerful” at locating a repairable since they target the entire prior TCU as a potential trouble‐source (Robinson, 2013). Once repair has been initiated in this way, an open class repair initiator leaves it entirely up to the speaker of the trouble‐source to identify and fix the problem through repetition, clarification or some other method of repair.

Where one method of repair fails, people tend to “upgrade” to incrementally more powerful methods until the repair is complete (Sidnell, 2011, p. 117). For example, using “who?” or “when?” or other category‐specific “wh‐words” can target more specific elements of the prior turn and provide a way for the speaker to identify what, in particular, the recipient is identifying as the trouble‐source. An even more specific variant of this format is a combination of a wh‐word along with a partial repeat of the prior turn such as “you said what?” This specifies the precise location of the trouble‐source within the prior turn by “cuing up” the specific element replaced by the wh‐word for repair. This “technology of repair” (Schegloff et al., 1977) in OIR works like the “cut‐off and re‐do” method used in self‐initiated self‐repair when a speaker stops then repeats the last few units of a turn before producing a replacement repair. The repeated units effectively “frame” the trouble‐source for the speaker to repair. An even more powerful format involves the recipient initiating repair by offering a candidate hearing or understanding for acceptance, rejection or reformulation by the speaker of the trouble‐source. This is the most specific or “powerful” form of other‐initiated repair because it explicitly displays what the recipient has heard or understood. This notion of “power” in repair formats can be placed on a scale from “weak” to “strong,” ordered on the basis of observed regularities in how people use one then upgrade to another to identify and repair troubles (Sidnell, 2011, pp. 117–118). For example, in Extract 12, Arthur begins by using an open class repair initiator when he says “Huh” in line 5 after June tells him she is going to ask the Krona margarine company to send her a box of replacement packages. When June does not provide an adequate repair, Arthur “upgrades” to a “partial repeat + wh‐word” in line 8, that is, using an OIR format with more power to locate the specific trouble‐source.

**Table:** 

**Extract 12** CABNC_KSS_074205_1		
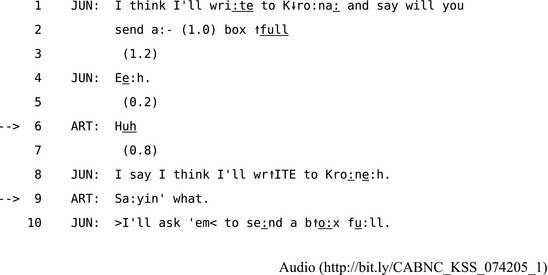	

**Figure 4:**
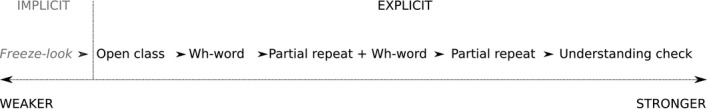
A spectrum of other‐repair initiation types (Clift, 2016; Manrique & Enfield, 2015a; Sidnell, 2011).

Fig. 4 presents this scale and includes the weakest known form of OIR based on recent studies of sign languages and gesture that show how a recipient suspending their movements in a “freeze‐look” can function as an “implicit” form of OIR that works as (or alongside) the positioning of this repair initiation in the next turn (Clift, 2016; Manrique & Enfield, 2015).

By tracking the detail of the repair methods people use in everyday situations, we can begin to see the specific misunderstandings that participants identify and resolve within a sequence. This episode begins after June has announced that she will write to Krona. She then pursues a response from Arthur in line 4. When he responds with “Huh,” she treats his OIR as indexing a problem of hearing by repeating part of her prior turn as the repair solution in line 8. However, in line 9, Arthur then upgrades his OIR, specifying more precisely what he still has not heard, to which June responds by re‐issuing that part of her prior turn in line 10. The more of the prior turn Arthur repeats the more precisely he can target the trouble‐source. The choices “others” make about how to initiates repair allows participants to display and track apparent states shared perception and mutual understanding. For example, in Extract 13, Nina talks to Clarence about the hedge in their garden.

**Table:** 

**Extract 13** CABNC_KBP_026503_1		
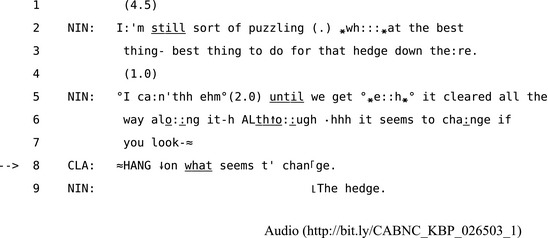	

Clarence's OIR in line 8 calls Nina's turn to a halt, then he uses a “wh‐word + partial repeat” to target the specific trouble‐source. Nina clearly responds to this specificity by producing only the repairable item “The hedge” in line 9. This is a minimal joint solution to a trouble because it only minimally disrupts the ongoing flow of interaction before progressivity is resumed.

Since other‐initiated repair is always a collaborative undertaking, some formats occupy the boundary between other‐ and self‐ repair in terms of who actually resolves the trouble in question. Candidate understandings, for example, which Schegloff et al. (1977, pp. 378–379) describe as a form of “other‐correction” involve a recipient offering a solution to the trouble‐source for the speaker to accept, reformulate, or reject. This procedure may be relatively cumbersome and may involve an extended expansion of the problematic sequence that involves both parties, to some extent, in finding a solution. For example, in Extract 14 Jessica tells her mother Sophie and Sophie's friend Cherrilyn about an arrangement she is making with a friend and a series of two candidate understandings are offered before one is reformulated and the other accepted.

**Table:** 

**Extract 14** CABNC_KBL_040301_1	
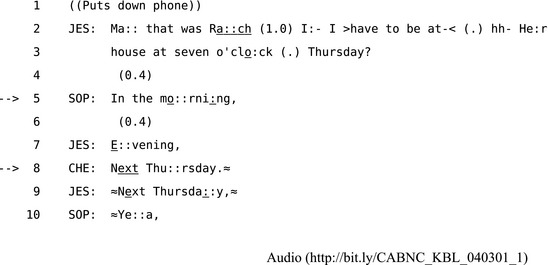	

In the first OIR at line 5 Sophie offers a candidate understanding of Jessica's arranged time as seven in the morning, which Jessica then rejects in line 7 by reformulating Sophie's candidate understanding from “morning” to “evening.” Then in line 8 Cherrilyn's request for confirmation “Next Thu::rsday.” provides Jessica with the opportunity to confirm and accept the candidate by repeating it in line 9 (Schegloff, 1996). Although these candidate understandings are more explicit, extended, and can delay the progress of the interaction more than other OIR formats, they also provide participants with stronger evidence of shared understanding and secure intersubjectivity. When people make arrangements for where or when to meet and misunderstandings may have inconvenient consequences, they can use these OIR “understanding checks” as a maximally secure—if cumbersome—way to confirm that intersubjective understanding has been achieved.^12^

### 2.6 Other‐initiated other‐repair

The least common form of repair is other‐initiated other‐repair (Schegloff, 2007, p. 101), where, for example, a recipient (other) responds while explicitly correcting something in the initial talk of the speaker. These actions are often done with displays of reluctance or hesitation. For example, when recipients initiate repair and provide a candidate solution, their talk is often delayed, disfluent, or accompanied by other marks of uncertainty such as “you mean,” “I think,” and tag questions like “isn't it?” (Schegloff et al., 1977, p. 378). This kind of hesitancy does not necessarily show that there is anything wrong or inappropriate with this method of repair. Rather, marking a turn with hesitations or delays is one method speakers can use to introduce uncertainty or doubt into whatever action is currently underway. For example, in Extract 15 Stuart's use of an other‐initiated other‐repair introduces some doubt and delay into Anne's suggestion about what they should eat for dinner.

**Table:** 

**Extract 15** CABNC_KB7_019202_1	
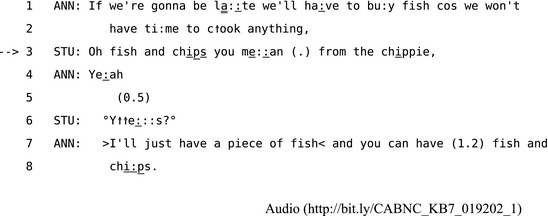	

This apparently mundane example of other‐initiated other‐repair contains far richer detail about what Stuart and Anne are doing in this episode of interaction than would be revealed by focusing primarily on the information exchanged between the parties. Stuart's repair initiation in line 3 provides a candidate understanding of Anne's dinner suggestion as a repair solution, and Anne then takes the opportunity to confirm it in line 4. It is worth noting that Anne's dinner plans in line 1 seem close to being settled as something they “have to” do. However, by offering Anne a candidate understanding of what “you me::an,” Stuart gives her the task of accepting or rejecting his repair solution. His modified repeat^13^ of her confirmation “°Y↑↑e:::s?°” at line 6 treats Anne's confirmation as insufficient, or at least that is how Anne treats it when she expands on her plan for the meal in lines 7 and 8. Without taking the analysis of Extract 15 any further, it is worth noting that Stuart's repair begins a process of renegotiation of the apparently settled dinner plans through which he eventually creates the opportunity for Anne to volunteer to cook instead.

Other‐initiated other‐repair seems relatively infrequent (Kitzinger, 2012, p. 231) compared to instances of self‐repair (Clift, 2016; Schegloff et al., 1977, p. 274). This rarity may be partly explained by recent quantitative studies by Kendrick (2015a) that show how the timing of OIR turns is systematically 400–600 ms slower than standard 100–300 ms turn‐transitions. This extra time provides several opportunities for recipients to avoid doing other‐initiation of repair because the speaker of a trouble‐source gains an extended opportunity to do self‐initiated self‐repair and the recipient has extra time to resolve problems of hearing or understanding themselves. The recipient also has time to produce visible displays of trouble such as facial expressions that can cue a trouble‐source speaker to initiate self‐repair. All these structural features of OIR may explain a general “structural dispreference” that mitigates against it and provides additional opportunities for other forms of repair initiation.

An even more unusual form of other‐repair involves what Schegloff et al. (1977) calls “other‐correction,” which explicitly corrects the previous speaker's trouble‐source. This correction of another's talk is typically followed by apologies or self‐corrections by the previous speaker in the next turn. Extract 16 is a canonical example of this type of other‐repair, which is often used when speaking to children or otherwise dealing with issues of speaker‐competence, pronunciation or other “correctable” errors.

**Table:** 

**Extract 16** A canonical example of ‘other‐correction’ from Jefferson (1987, p. 87)	
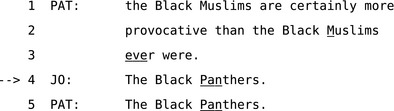	

Recent studies have used this distinction for coding and quantification (Kendrick, 2015a,b) and have found that other‐corrections are often produced without the long delays associated with most other forms of OIR. Schegloff et al. (1977) suggest that other‐correction is often used in parent–child or similar interactions that involve instructing someone not yet competent in self‐monitoring and self‐repair. Forms of other‐correction where a recipient treats a speaker's talk as incorrect and needing immediate repair may be done more quickly than the delayed, disfluent OIR procedures that recipients tend to use when dealing with more delicate or equivocal problems of hearing and mutual understanding.

### 2.7 Repair as a starting point for analysis

Repair is a procedural “self‐righting mechanism” for problems of speaking, hearing or understanding in talk (Schegloff et al., 1977). To solve these problems, people have to bring them to the surface level where we see them working together to achieve sufficient mutual understanding to move on. Because repair operates at this surface level, it provides analysts with empirical footholds at a vantage point within the participants’ world where the sequential structure of each repair highlights whatever the participants are currently treating as relevant and mutually understood—or not—in any given situation. This makes repair a useful starting point for exploring other conversational procedures. For example, people often use repair or disfluency to do “pre‐disagreements” (Schegloff et al., 1977; note 28) that can foreshadow or even “head off” having to do more explicit forms of disalignment or disagreement.^14^ Similarly, self‐repairs can provide additional insight into otherwise tacit episodes of near‐misunderstanding or near‐disagreement (Schegloff, 2010), whereas an “untroubled” utterance would have given away no further information. Conversely, observing how participants deal with other‐initiated repair can expose conventions about people's rights and obligations to correct one another's talk in specific situations even if these ostensible “rules” themselves never become explicit (Bolden, 2018). The repairs analyzed here showcase just a few of the primary distinctions between methods of repair documented by CA over the last half century. Studies of repair offer many useful measures and methods for asking cross‐disciplinary research questions about shared understandings that extend far beyond the local structure of a sequence of talk. For example, corpus‐based and experimental studies have studied the effects of various factors on repair procedures (Bortfeld, Leon, Bloom, Schober, & Brennan, 2001) and have shown how people achieve an incrementally shared “common ground” of information (Clark & Brennan, 1991) in order to achieve shared understanding. This area of research in psycholinguistics, formal semantics and pragmatics has successfully operationalized disfluencies (Brennan & Schober, 2001; Clark & Fox Tree, 2002; Ginzburg et al., 2014; Oviatt, 1995), requests for clarification (Ginzburg, 2001; Healey, Plant, Howes, & Lavelle, 2015; Healey et al., 2003; Mills & Healey, 2006) and repair in general for computational models of dialog (Hough & Purver, 2017), and innovative experimental designs in dialog research (Healey, Mills, Eshghi, & Howes, 2018). CA itself does not study mental states, intentions, or cognitive processes since these are not directly accessible to participants in interaction at the surface level of talk (Clift, 2016, p.234). However, repair is clearly a useful concept for the study of cognition and some more recent developments in CA are starting to find methodological meeting points with cognitive science.

## 3 New interfaces between cognitive science and conversation analysis

CA has tended to eschew coding and quantification (Schegloff, 1993) in order to avoid premature theorizing and the necessity of reducing its interactional phenomena for experimental study (Kendrick, 2017). Because social interaction occurs between individuals in specific social situations, it can be methodologically problematic to categorize, “code”, and count behaviors as specific “types” of action for quantitative analysis (Stivers, 2015). CA has therefore tended to use inductive, observational forms of generalization such as building collections of cases containing hundreds of recorded clips of related interactional phenomena and subjecting them to repeated qualitative analysis (Hoey & Kendrick, 2017; Schegloff, 1996) and informal peer review via group “data sessions” (De Ruiter & Albert, 2017). However, recent developments suggest that certain types of repair can be very amenable to coding and quantification for statistical analysis (Dingemanse, Kendrick, & Enfield, 2016). Although there is a clear methodological and philosophical distinction between the descriptive methods of CA and formally testable experimental hypotheses (Nishizaka, 2015) these distinct approaches can be—at the very least—mutually inspiring (Steensig & Heinemann, 2015) and may—at best—lead to new and much‐needed interfaces between conversation analysis and experimental methods in interaction research (De Ruiter & Albert, 2017).

### 3.1 Quantification in CA, from turn‐taking to repair

Initial approaches to using CA in conjunction with experimental and statistical methods have focused on core findings about turn‐taking (Sacks et al., 1974). For example, Wilson & Zimmerman (1986) were amongst the first to operationalize and test predictions based on CA's turn‐taking system against stochastic and signaling models for the distribution of turns in conversation. These studies have supported hypotheses based on CA's model across at least 10 different languages (Stivers et al., 2009). CA findings have also informed experimental studies of whether structurally “preferred” responses in interaction, such as responding to an invitation by accepting rather than rejecting it, are done faster than “dispreferred” responses, such as rejecting invitations or offers (Kendrick & Torreira, 2014). Similarly, by testing the timing and construction of turns at talk researchers have been able to test CA‐informed theories about the planning and projection of turns and the cognitive/linguistic architecture that presumably supports these processes (Levinson & Torreira, 2015).

More recent quantitative CA studies have begun to operationalize various features of repair. For example, descriptions of self‐repair can be tested using models and predictions that study how speakers of different languages will do repair given the specific prosodic and morphsyntactic resources available to them (Clift, 2016, pp. 237–239). In one such study, Fox et al. (2009) categorized, quantified, and analyzed various forms of SISR across seven different languages and showed how language‐specific resources—different words and syntactic formats—systematically affect the specific position where speakers most often self‐initiate repair. Related studies of SISR—both qualitative and quantitative—also suggest a close association between linguistic resources and patterns of repair‐initiation and self‐repair across very different language groups (Fox, Maschler, & Uhmann, 2010; Laakso & Sorjonen, 2010). Quantification of self‐repair may avoid some of the methodological complexities of coding interaction because it is produced by a lone speaker and—when it occurs naturally—it clearly constitutes something relevant to the speaker in any given situation because it involves stopping and fixing something before progressing with a stream of talk.

Other‐initiated repair may seem relatively complex because it involves multiple parties and sequences. However, the easily recognizable structure of OIRs (especially in the turn following a trouble‐source) makes them particularly identifiable and therefore useful for large‐scale quantitative studies (Kendrick, 2015a). They are also useful for quantification because they are so common. While emphasizing that distributional findings alone cannot explain interaction, Schegloff (2000, pp. 210–211) reports that 90% of other‐initiated repairs in his corpus of 350 instances are initiated in the turn following the trouble source, while Kendrick (2015b) finds 81.5% next turn OIRs out of a corpus of 185 cases.^15^ In their study of the word “huh?” across 21 languages, Enfield et al. (2013) suggest that this is possibly the most generalized form of other initiated repair. The remarkable consistency of how people not only pronounce and use “huh” across 10 languages (Dingemanse et al., 2013)—but also the subtle variations in phonetic form and intonation—suggest it may be a “universal word” that is shaped by interrelated environmental, cognitive, and interactional constraints. The timing of repair and its intersection with the turn‐taking system also provides a useful basis for experimental predictions. In a quantitative study of OIR formats Kendrick (2015a) shows that the timing of other‐initiation turns are most frequent after a gap of 700 ms. This means that other‐initiated repair seems to produce a 400 ms delay relative to the standard 100–300 gap length of the most common non‐OIR types of turn‐transition in copresent talk.^16^ Because of its intersection with the turn‐taking system, the timing of OIR can show how people balance the competing requirements of progressivity and intersubjectivity in conversation (Benjamin, 2012; Heritage, 2007). Repair is clearly a high priority activity for participants in interaction since they suspend whatever else they are doing until it is complete (Sacks et al., 1974, p. 720). The way repair interacts so systematically with conversational turn‐taking shows why it can be such a useful phenomenon for approaches to quantification and generalization in interaction research (Dingemanse & Enfield, 2015).

### 3.2 Repair beyond talk

CA research is increasingly focused on non‐vocal bodily action including sign language (Manrique, 2016; Manrique & Enfield, 2015a; Willoughby, Manns, Shimako, & Bartlett, 2014) as part of a more general move beyond its initial focus on repair as a talk‐related phenomenon (Schegloff, 2000). This broader “embodied turn” in interaction research (Nevile, 2015) challenges the “verbal or visual reductionism” (Mondada, 2016) that is a necessary for coding observations for quantification and experimentation. It also stretches the limits of existing systems for transcribing and categorizing collections of phenomena using conventional CA methods, which is leading to many novel and promising developments in this area of research. For example, in her procedural descriptions of repair in operating theaters Mondada (2014b) highlights how surgeons cut‐off their talk and become disfluent or hesitant when coordinating multiple concurrent courses of action during surgery (Haddington et al., 2013). These multiple and often simultaneous courses of action are not necessarily organized in relation to a system of turn‐taking (Sacks et al., 1974). They may therefore require specialized forms of multimodal transcription and analysis (Mondada, 2014a). Taking into account gesture, gaze, and bodily orientation (Floyd, Manrique, Rossi, & Torreira, 2014; Goodwin & Goodwin, 1986) and tactile or rhythmical interactional resources (Albert, 2015; Keevallik, 2010; Weeks, 1985) requires new approaches to transcription and coding because these behaviors are often simultaneous rather than turn‐constrained, and may use specialized forms of organization (Raymond & Lerner, 2014).^17^ Researchers are currently finding new ways to transcribe and analyze the distinctive methods and resources people use for repair—including both embodied and computer‐mediated interactions (Healey et al., 2003; Meredith & Stokoe, 2014; Schönfeldt & Golato, 2003)^18^. CA researchers are also finding new approaches to moving from single‐case analyses and the turn‐organized structures of talk‐in‐interaction toward wider generalizations, new contexts and new tools for studying repair in human interaction (Kendrick, 2017; Stivers, 2015). Just as CA research into repair in talk has facilitated computational and experimental studies of spoken dialog, studies of embodied repair have many methodological and theoretical meeting points with related studies of gesture and multimodality in cognitive science (Healey et al., 2015).

### 3.3 Conclusion: The interface between interaction and cognition

Repair can function as a theoretical interface for cross‐disciplinary research between CA and cognitive approaches to human interaction. This paper has shown how the methods of repair documented by CA can inform studies from multiple research perspectives, and CA can be mutually inspired by developments in related but methodologically distinct fields. On the CA side of this interface, methodological meeting points with forms of quantification and experimentation are already underway. On the other side of this notional interface, CA's studies of repair have already inspired a growing body of research that has successfully operationalized various forms of repair for experimental and corpus‐based studies of dialog and human interaction. These converging lines of research lend support to two assertions we believe are complementary, but that have more often been pitted against one another (De Ruiter & Albert, 2017). It is useful and necessary to recognize and clarify the striking epistemological and methodological distinctions between CA's minimal approach to shared understanding and various experimental or information‐based approaches.^19^ At the same time, we argue that repair can work as an interface between these approaches and offers many opportunities for mutual inspiration and collaboration. The forms of repair documented in this article aim to provide researchers in the cognitive sciences with a primer for engaging in this kind of cross‐disciplinary study. Examples of each major type of repair here include links to openly licensed audio data from the AudioBNC which can be downloaded, examined independently, checked, and re‐analyzed.^20^ Having provided resources for engaging with repair, we conclude with some ideas as to how this kind of cross‐disciplinary research could work in practice by outlining some key opportunities and challenges, and pointing to broader research questions at the interface of conversation and cognition.

Firstly, CA's orientation to detail and descriptive sensitivity in describing and categorizing repair can offer new insights and operationalizations to theory‐led approaches to interaction research. On one hand, the repair methods outlined here show how CA can uncover structure and levels of organization that would be very hard to theorize about (Heritage, 1984b, p. 311) if they had not been discovered and documented over decades of systematic inductive observation of interaction. On the other hand, CA's procedural descriptions highlight the specific challenges of coding, quantifying, and experimentally manipulating interaction. Understanding these analytic constraints can also, conversely, highlight interactional phenomena that *are* more amenable to coding, quantification, and experimentation without either compromising the ecological validity of the phenomena or losing sight of them altogether.^21^ There are corresponding opportunities for CA researchers to engage in cross‐disciplinary studies of repair that test cognitive theories and computational models of dialog. This paper has argued that a common focus on repair can guide research questions that reach beyond the surface level of talk. Mental representations, cognitive processes, and theoretical constructs are not addressed directly by CA due to its methodological constraints on theorizing about anything that is not clearly drawn into an interactional situation by the behavior of participants themselves. However, the ripples on the surface of talk created by repair do reveal how people work to understand and to make themselves understood in ways that involve endogenous theorizing about and tracking others’ displays of understanding. For example, when a speaker initiates third position repair after the recipient's response reveals a problem with their initial turn, this can be seen as a kind of cognitive theorizing on the part of the speaker themselves. In order to successfully achieve repair and move on with the interaction, the speaker must engage in tracking the recipient's ongoing understanding based on whatever evidence breaches the surface level of talk.

Among CA's phenomena, misspeaking, mishearing, misunderstanding, and the repair procedures people use to deal with them offer particularly useful moments for analysis. When someone encounters trouble in talk that—in order to proceed with the interaction—requires resolution, they work to display what kind of trouble it is, and then work together to resolve it to the point that they can move on. Repairs are rich in opportunities for analysis because they are inherently analytical moments where the specific kind of trouble at hand may be an ostensibly cognitive problem of mutual understanding, a practical problem of speaking or hearing, or a contextual problem of propriety or transgression. When these moments occur, they open up opportunities for inspection of the current situation from multiple perspectives. Participants in the situation can interpret and respond to the sequence and organization of the repair based on who initiated the repair, when, and how they initiated it. Conversation analysts can review recordings of the repair multiple times, and can slow down and process the recording to develop analyses of what kinds of repair procedures the participants are using. Researchers in the cognitive sciences can then draw on CA to code, quantify, and draw inferences from aggregate statistics, or take inspiration from these moments to develop new experimental designs. The repair methods we have for identifying and dealing with occasions of apparent miscommunication are the very structures that uphold the possibility of organized communication and coordination in the first place. It makes sense, then, that to converge on a shared understanding of interaction from a variety of methodological perspectives, we should use repair as a vantage point from which we can study how interaction breaks down, and how people cooperate to shore up the foundations of human social action.

## Notes

1 More generally, as a branch of ethnomethodology (Garfinkel, 1967)—the study of the methods that reflexively constitute the recognizable norms, membership and protocols of any social group—CA tends to avoid theorizing about mental states and uses forms of evidence that are demonstrably available to participants in interaction.

2 For example, Schegloff (1993, p. 104) criticizes a study of the frequency of laughter compared to the frequency of other “backchannels” (Yngve, 1970) by explaining that “parties do not laugh per minute. Laughter is amongst the most inescapably *responsive* forms of conduct in interaction.” The “denominator problem,” as he calls it, here comes from the mistake of measuring the occurrence of an interactional behavior “per minute” rather than in relation to the number of jokes or other opportunities an interlocutor might create for others to laugh in a given interaction.

3 CA's foundational paper by Sacks et al. (1974) is entirely devoted to a detailed description of the turn‐taking system.

4 Note that audio‐only recordings of co‐present interaction seriously limit the scope of analysis. CA began by studying telephone calls (where having audio‐only data was not an analytic limitation since both participants and analysts shared access to both sides of the call) and today conversation analysts usually insist on using video data to study copresent interaction.

5 Note that a repair space such as the request for clarification in lines 2–3 of Extract 3 may be “nested” within other sequences of action (e.g., where an answer to a question also initiates repair). This highlights the importance of maintaining a distinction between the numbering of turns in a series of utterances and the numbering of positions in a sequence of actions during a sequential analysis.

6 There is also “fourth position repair” but this is relatively infrequent and less often analyzed, although Schegloff (1992) outlines how it works: After a three‐part initiation, completion, and acknowledgment sequence, where the acknowledgment reveals trouble with the acknowledged understanding, recipients may then initiate repair from this fourth position in a sequence.

7 Of course, within other analytical approaches to interaction, repair is still very useful as a way to operationalize informational “grounding,” “alignment,” or “convergence/divergence” in dialog (Clark & Brennan, 1991; De Ruiter, 2013; Healey, Purver, & Howes, 2014; Pickering & Garrod, 2004).

8 See Extract 15 for an example of this kind of issue where initiating a repair opens up the opportunity for one party to dissemble and renegotiate an apparently settled joint decision.

9 A self‐initiated self‐repair can also be analyzed as a “third turn repair” (Schegloff et al., 1977) when, for example, a recipient produces only a minimal response such as “mhm”—sometimes called a “continuer” (C. Goodwin, 1986; Schegloff, 1982)—in the second turn. A self‐repair by the speaker at this point then occupies the third turn in the sequence. Continuers are just one of the phenomena that Yngve (1970) lumps together as “backchannels.”

10 De Ruiter (2006) has suggested that one of the reasons speakers often gesture during a search is either to “keep the floor” or to invite the listener to assist in the search by providing the looked‐for item. This runs counter to the earlier suggestion by Krauss and Hadar (1999) that producing the gesture serves the speaker by facilitating the search process itself.

11 Of course, there is always the possibility that participants might, at any point in the future, notice that there has been some problem in earlier interaction and then stop what they are doing and address the breakdown explicitly. However, this would constitute a new sequence of action that would be structurally distinct from whatever was underway when the trouble arose. Repair, in CA terms, refers only to interactional work undertaken within the “repair space” opened up within a current sequence of action.

12 Studies of how people manage person and place reference in conversation suggest that participants constantly balance the competing demands of progressivity against the imperatives of maintaining security of intersubjectivity (Heritage, 2007; Stivers, Enfield, & Levinson, 2007).

13 Stivers (2005) describes a similar method whereby recipients do a “confirmation check” as a way of displaying and managing their respective rights and obligations to confirm or query the action underway, which in this case involves making dinner plans.

14 Extract 15 shows a good example of a repair with which Stuart “unsettles” plans to buy fish and chips, and begins the delicate matter of letting Anne volunteer to cook instead.

15 This difference of 8.5% may be partly explained by Kendrick (2015b) excluding common markers like “ritualized disbelief” and news receipts such as “you did?” or “really?” from his corpus.

16 These kinds of timing measures can reveal other subtle procedural regularities in different repair formats. For example, OIR formats that locate the trouble‐source in the prior turn tend to be produced faster than open class repair initiations such as “huh?” and OIOR appear to be faster still.

17 For example, even the earliest studies of gaze behavior (M. Goodwin & Goodwin, 1986) showed that when searching for a word, people can equivocate as to whether they are doing self‐initiated self‐repair or are self‐initiating and inviting other‐repair by shifting their gaze towards or away from the recipient in particular ways. Embodied repair and especially the kinds of subtle, designedly ambiguous actions that can be accomplished using repair constitute an exciting set of challenges for CA's existing methods, technologies, and transcription systems.

18 This article is primarily focused on a survey of repair, which has mostly dealt with talk‐in‐interaction, so there is no space for an extended discussion of various forms of mediated communication and the affordances they offer for repair. However, see Arminen, Licoppe, and Spagnolli (2016) for a recent review of mediated interaction in CA, and Meredith (2017) for a recent discussion of how to adapt CA's transcription system for the dynamics of online text‐based interaction.

19 For a full discussion of these distinctions in relation to the philosophy of science, see De Ruiter and Albert (2017).

20 Any re‐transcriptions of these data should be contributed back to the open data CABNC corpus (Albert, De Ruiter, & De Ruiter, 2015).

21 See De Ruiter (2013) for a discussion of ecological validity in interaction research, and Garfinkel (2002, pp. 264–267) for a clear warning about “losing the phenomenon.”

## Appendix A Conversation analytic transcription conventions

### A.1

**Table:**

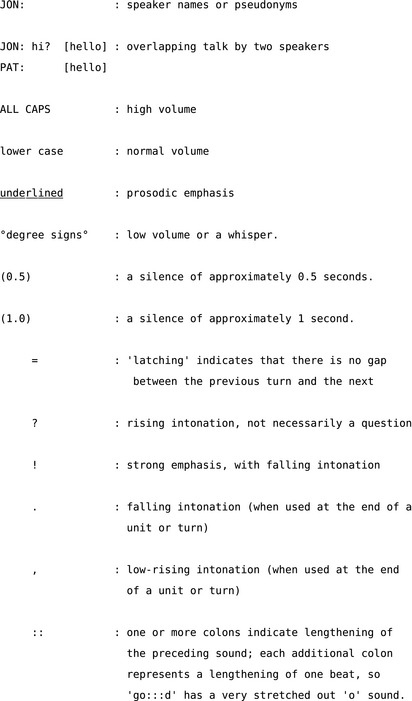

**Table:**
